# Comparing South Africa’s Sustainability and Circular Economic Roadmap to The Rest of the World

**DOI:** 10.1007/s42824-023-00073-x

**Published:** 2023-03-22

**Authors:** Mohamed Sameer Hoosain, Babu Sena Paul, Wesley Doorsamy, Seeram Ramakrishna

**Affiliations:** 1 University of Johannesburg, Johannesburg, South Africa; 2 University of Leeds, Leeds, UK; 3 National University of Singapore, Singapore, Singapore

**Keywords:** Circular economy (CE), Fourth industrial revolution (4IR), Sustainability, Environmental, Social and governance (ESG), United Nations Sustainable Development Goals (UN-SDGs)

## Abstract

In 2015, the United Nations Member States developed a collective blueprint for sustainability and development. The 2030 Plan includes the 17 UN-SDGs, which are an immediate call for action from all countries in the form of a global collaboration. To date, a number of countries have made significant strides in achieving the goals. One solution is the transition from a linear economy to a circular economy. Together with this, new 4IR innovative technologies has helped many countries in their transition to a circular economy as well as achieving the SDGs. Countries and organizations have also adopted environmental, social, and governance reporting as another technique, and have become mandatory in some regions and organizations. The economy of South Africa is beset by poverty and inequality, considerable unemployment, carbon-intensive, water insecurity and slow GDP growth. Furthermore, the COVID-19 epidemic has caused the economic crisis to worsen further and emphasizes the need for a new development strategy to spur economic recovery. In this paper, we will compare South Africa’s sustainability and circular economic road map to the rest of the world, and we suggest solutions and policies that can be put in place for the future benefit of the country.

## Introduction

In September 2015, the United Nations General Assembly adopted the 2030-Agenda for Sustainable Development, which includes 17 Sustainable Development Goals. The new agenda emphasizes a comprehensive approach to attaining sustainable progress for all, based on the notion of “leaving no one behind.” They are classified into three types of systems: environmental, economic, and social. There has been a number of newer solutions being developed around the world to achieve the goals in different sectors. Most recently concepts such as 4IR technologies and transitioning to a Circular economy are being used to achieve the SDGs. Current circular approaches and research have shown that strong relationships exist between GOAL 6 (Clean Water and Sanitation), GOAL 7 (Affordable and Clean Energy), GOAL 8 (Decent Work and Economic Growth), GOAL 12 (Responsible Consumption and Production), and GOAL 15 (Life on Land) (Schroeder et al. 2018).

The global demand for resources is predicted to double by the year 2050. With this, it is said that critical resources will be depleted. It is clear that resources need to be handled more sustainably. Just 6% of materials are actually recycled (Haas et al. 2015). Industries across all sectors, and consumers have to reconsider their approach to resource management. Tonnes of aluminum, silicone and plastic are used to make a laptop that weighs a few kilograms. Every year 8 million tons of plastic is dumped in our oceans. Greenhouse gas emissions (GHGE) are spiralling out of control, and not forgetting our wasteful nature when it comes to water (Andrew 2018). These are just a handful of examples on our Take-Make-Dispose nature. Therefore, circular practices can be applied as a toolbox to solve issues like the ones mentioned above, while at the same time achieving a number of UN-SDGs.

To date, a number of countries have made significant strides towards adopting circular approaches. Together with this, new technological innovations such as; Artificial intelligence (AI), The Internet of Things (IoT), Big Data, Robotics etc. has helped many countries in their transition to a circular economy as well as achieving the SDGs (Hoosain et al. 2020a). Furthermore, countries and organizations have adopted environmental, social, and governance (ESG) reporting as another technique, some have even made this type of reporting mandatory.

We had selected South Africa (SA) as part of this research as the main authors originate from this country, hopefully this research and suggestions can be used for the benefit of the country in the near future. SA is a developing country, and is located on the southern tip of Africa. It is a country surrounded by several eco-systems, home to a number of ethnic groups, and filled with a number of public, private, and governmental sectors. The African continent has always been the last frontier when it comes to adopting newer approaches and technologies. In this paper, we will compare South Africa’s sustainability and circular approach to other countries around the world, so that suggested solutions and policies can be put in place.

## Background

### Circular Economy

The concept of a circular economy has gained momentum over the years both among academics and practitioners. Critics argue that it means a number of different things to different people. Research reveals that the circular economy is most frequently defined as a mixture of minimizing, reusing and recycling practices, whereas it is often not pointed out that circular thinking requires a structural change (Kirchherr et al. 2017). It gained interest in the late 1970s, with much literature suggesting a connection between sustainability and the circular economy. Organizations like the Ellen Macarthur Foundation have been promoting the concept across different partnerships and sectors.

Establishing a circular economy is a must to overcome increasing waste barriers, which are important for both protection of resources and the environment, and can contribute to a sustainable and competitive economy. The purpose of a circular economy can be described in three points (Ghomi et al. 2021):

- Maximizing the distribution of useful products in industries.
- Modifying usage trends and paying attention to recycled products rather than raw materials.
- Reducing the generation of waste by re-use and reducing the output of dangerous components in the environment.

The circular economy seeks to regenerate wealth, be it financial, industrial, human, social or environmental. It allows an increased distribution of goods and services. A circular economy provides environmental and economic advantages. Environmentally—reduces GHGE, builds critical eco-systems, and protects biodiversity. Economically—resources are being saved, economic growth, job creation, and increased demand.

**Fig. 1:**
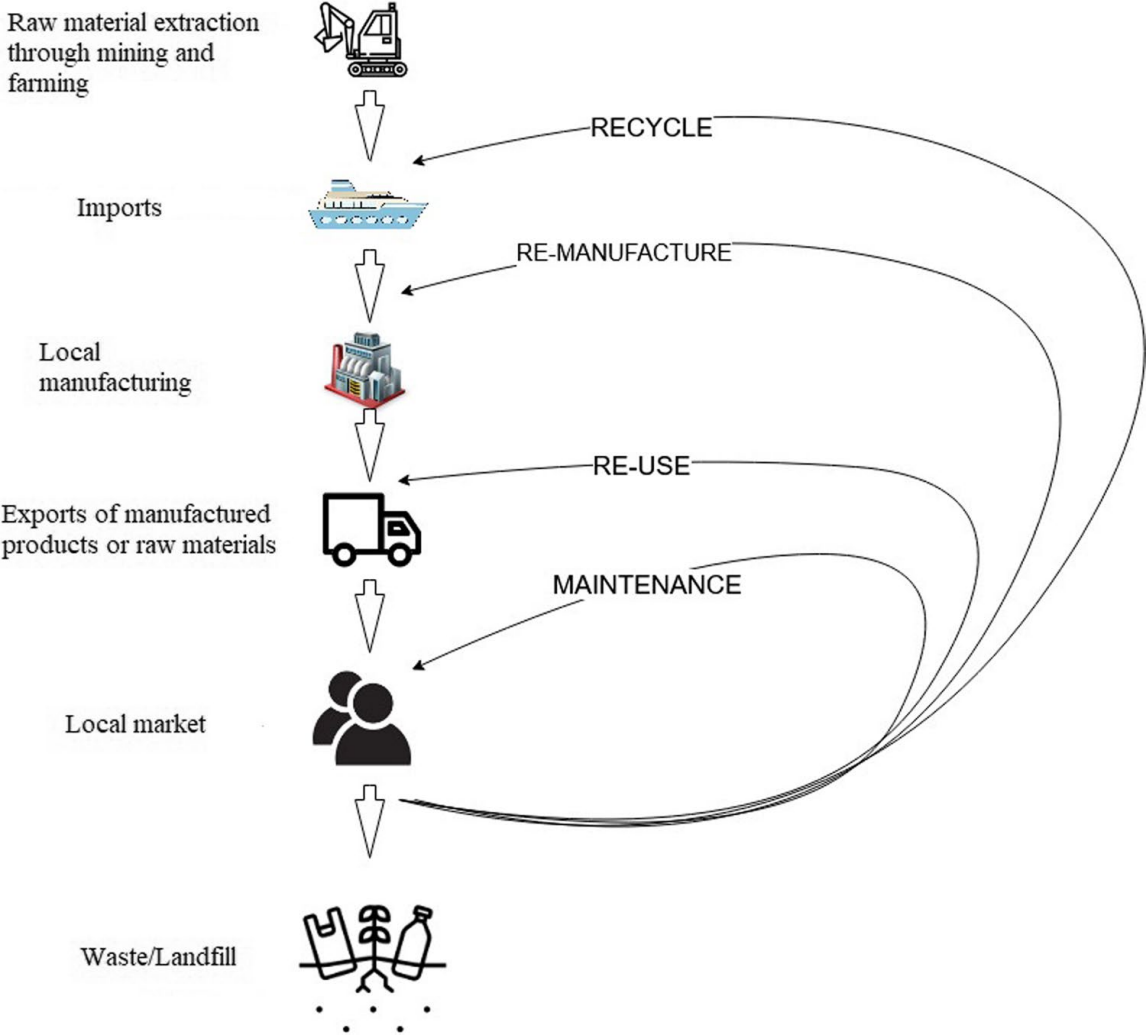
A typical South African circular economy approach

South Africa, a developing nation with abundant natural resources, is distinguished by its extractive-based economy, which is defined by high resource throughput, much of which becomes waste and a low rate of resource productivity. When resources are mined domestically, a significant amount of them are exported for further processing offshore while little is used to increase local stocks. A typical circular economy approach is shown in Fig. 1 by Kottaridou and Bofylatos (2019). The figure was remodelled from the famous Ellen Macarthur Foundation “Butterfly” diagram. The diagram shows the continuous flow of the technological materials around the “value circle.” We had further remodelled the diagram in a South African context.

Circular economy includes making use of existing assessment methodologies and tools. There are a number of tools that can used, and these have been made easier with the use of digital technologies. Digital technologies are in the form of online databases, software models, online calculators, and algorithms. Some of these tools are listed and explained in Table 1 (Hoosain et al. 2020a, b):

**Table 1:** Circular economy assessment methodologies and tools

Tools	Description
Material passports	Material passports is a value monitoring mechanism that can be used to bring back residual value to the market. Material passports make details about materials, substances, or systems readily accessible at any point i.e. from production, to purchasing, to usage, and to maintenance. The necessary information includes the material’s physical and chemical qualities, safety data sheets (MSDS, TDS), a bill of materials (BOM), logistics, disassembly, and finally recycling potential. The method of making one includes various organizations and stakeholders. The most popular passport databases are Madaster and Buildings as Materials Banks (BAMB)
Life cycle costing (LCC)	Derived from The International Organization for Standardization (ISO) 15,686. LCC is an economic technique that measures the cumulative cost of a product or resource, process or service, discounted over its lifetime. LCC is used for decision-making in different areas and for a number of purposes. There are three categories that it falls under: conventional, environmental, and societal
Life cycle assessment (LCA)	Derived from ISO 14040. LCA assesses the total environmental effects of the manufacturing process, use, waste and operations related to the design and maintenance of a building or a commodity or material. However, economic or cultural considerations are not taken into account
Social Life Cycle Assessment (S-LCA)	S-LCA adheres to the ISO 14040 framework; nevertheless, some features change, are more prevalent, or are intensified at each stage of the research. S-LCA does not provide information on whether or not a product should be created, but can provide information that is useful in making a choice. The United Nations Environment Programme (UNEP) is in charge of coordinating environmental responses across the United Nations system. They have also suggested recommendations and procedures for creating life cycle inventories
Material Circularity Indicator (MCI)	MCI is a decision-making mechanism intended to assess how well a company or commodity performs in the transition from a linear to a circular economy. The value of the component or components MCI is between 0 and 1 (or 0–100% of the recirculated parts), a value that is greater than 1 means a higher circularity. Due to the complexity of the MCI calculation, there are a number of online digital calculators available, including the Circular Economy Toolkit (CET), the Circularity Calculator, the Material Circular Indicator (MCI) tool developed by Ellen MacArthur, Flex 4.0 by the Delft University, and RELi 2.0 by the USGBC, to name a few

### Circular Economy and Industry 4.0

We have witnessed an increase in technical advancements since the start of the industrial revolution. When electricity was introduced in the twentieth century, manufacturing grew. Finally, we saw automation in the 1970s. Initially, factories were powered by water and steam engines in the nineteenth century. We are currently on the verge of an emerging digital industrial technology called Industry 4.0. (Hoosain et al. 2020a). In today’s media, 4IR-related technologies are typically depicted in a dystopian light as the ones that will steal our employment or possibly even take our lives for the wrong reasons. However, it may also turn into a potent tool in the global effort to achieve the SDGs.

Therefore, in order to improve circular economy plans and practices and address environmental problems, digital technologies are crucial. The ideas of the Circular Economy and Industry 4.0 are connected. Technology has a role at the producer, consumer, and policy levels in the circular economy by closing and tightening the material flow loop. Internet of Things, Cloud Computing, Cybersecurity, Big Data, Robotics, Additive Manufacturing, Augmented Reality, AI, and other technologies are all part of Industry 4.0. Additionally, the construction of a track record for items and their components is made possible by blockchain technology. The information flow needed to enable the creation of a circular economy is made possible by the pairing of blockchain information with actual physical things. AI could make it easier for designers to think about a product’s capabilities, reusability, reparability, and durability. An autonomous technique of sorting waste seeks to decrease waste and pollution, and AI plays a significant part in a successful waste management system. In order to improve waste management systems in many nations, cutting-edge technologies including Radio Frequency Identification (RFID), IoT, and Sensor Networks (SN) have been deployed. The nexus of the Circular Economy and Industry 4.0 creates a new paradigm for the use of natural resources in SDG success initiatives (Khajuria 2021).

In South Africa, farming and mining are huge sectors, the concept of circular economy is not new to these sectors. With the introduction on these new digital trends it may result in *smart farming* or *smart mining* with more cutting-edge, technological, and environmentally friendly procedures, goods, and services. The idea of sustainability and circularity can be fast tracked with the use of these innovative digital tools.

### Environmental, Social, and Governance

**Fig. 2:**
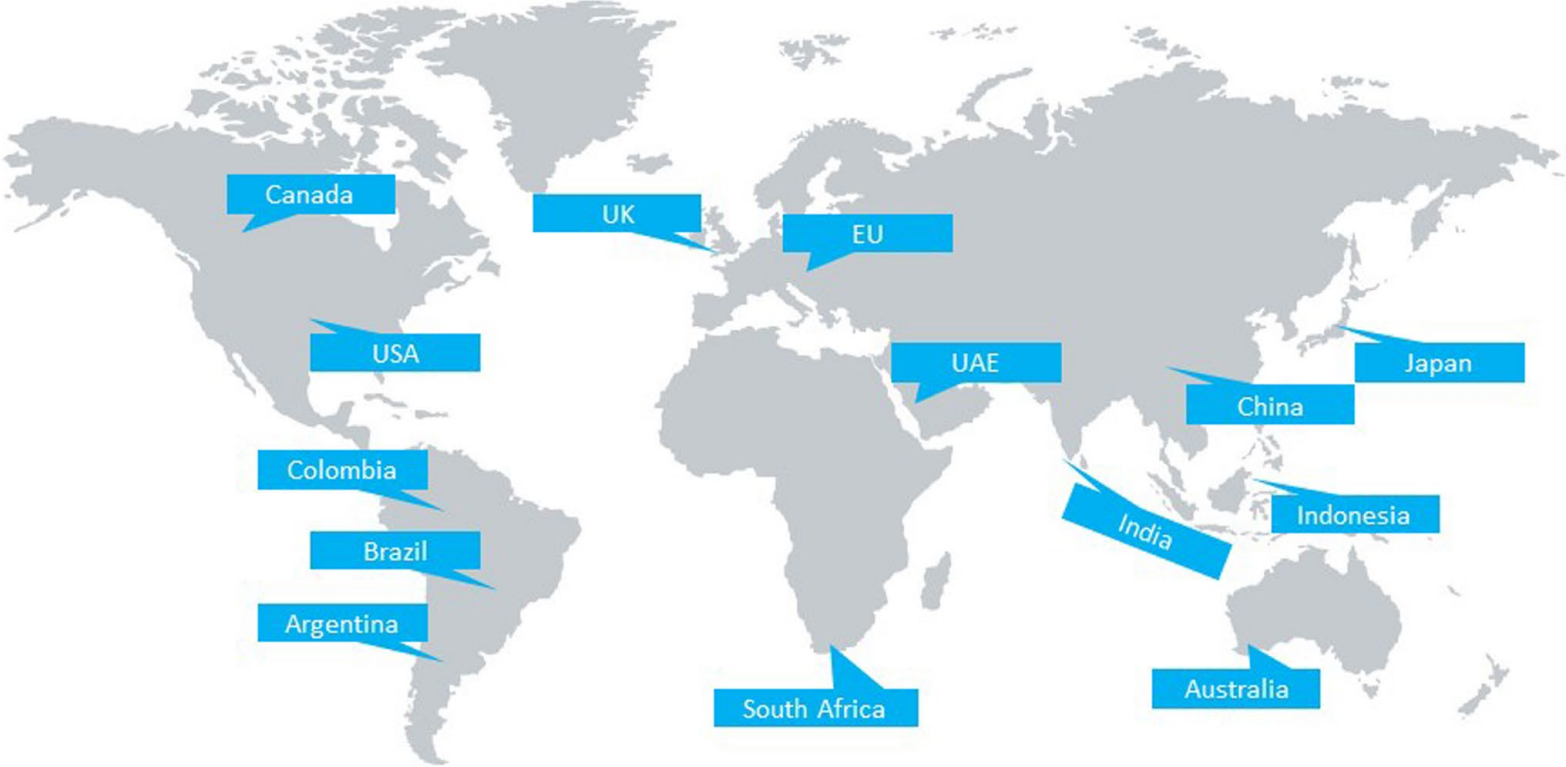
Some of the countries with the highest GDP’s that are implementing reporting can be seen here. Carrots and Sticks have progressed from covering 19 countries in 2006, 32 countries in 2010, 45 countries and regions in 2013, to 50 countries and regions in 2016. By 2016, they managed to cover the 50 biggest economies (by GDP) in the world. The latest full list of countries and reporting instruments can be found at https://www.carrotsandsticks.net/ (Van der Lugt et al. 2020)

ESG is one of the three primary determinants of the sustainability and social implications of a company investment. This has been made mandatory in recent times by a number of countries. The most used list for ESG is the UN-SDGs. Similarly, ESG documentation brings back value and acts as an empowering mechanism towards sustainability, for market processes to push circularity, and to remedy the existing shortcomings of the linear economy. ESG reporting has become important in recent years in promoting the shift in sustainability around the world and especially for investors. Big corporations like Unilever, Apple and several more companies are now publishing metrics to make sure consumers align them with sustainability. We list and describe the top companies implementing ESG in Table 2, and in Fig. 2 the countries with some of the highest Gross Domestic Products (GDPs) that are implementing reporting (Van der Lugt et al. 2020; AlphaSense Staff 2021).

**Table 2:** Some of top companies that are implementing ESG reporting

Tools	Description
NextEra Energy (NEE)	NextEra is the biggest electrical utility by cap on the market. Over the past few decades, the organization has steadily reduced its emissions through increased use of renewable energies
NVIDIA (NVDA)	NVIDIA, best known for its graphics cards, is a highly regarded ESG corporation and it has a strong stance on conflict minerals. The organization has special due diligence protocols to ensure that it never includes conflict minerals in its goods
Microsoft (MSFT)	Microsoft has taken the forefront in its contribution to climate reduction by being the first organization of its peers to target carbon negative status by 2030
Salesforce (CRM)	Salesforce has several significant goal-based ESG programs in place, it has dedicated 1 million working hours to UN-SDGs
GlaxoSmithKline (GSK)	This UK-based pharmaceutical giant has a range of enormous ESG projects. A total of 13 commitments have been made that relate to the numerous UN-SDGs. The business intends to reduce its environmental footprint by 25% by 2030

According to the UN Environment Program, South Africa’s financial sector is a leader and an innovator when it comes to ESG implementations. Their regulations have been a major role player in this process. Environment concerns, governance and social aspects are huge concerns in South Africa, therefore the Financial sector conduct authority (FSCA) has provided guidance within South Africa’s regulations so that consideration with respect to ESG is integral when evaluating the sustainability of assets. Similar considerations are made for things like carbon-tax, minimum wage and energy efficiency (Group 2023).

## Research Methodology

A literature review was undertaken to investigate similarities and to establish useful evidence. The analysis process used in this article was accompanied by a snowballing technique for an in-depth assessment. We had searched and found a series of papers that concentrate on circular economy and sustainability around the world. The analysis was undertaken on scholarly papers (e.g. peer-reviewed journal articles and conference papers) and non-academic papers (e.g. government policy manuals, surveys, circular economic publications, sustainability publications such as the United Nations, and statistics) (Carrière et al. 2020).

## Literature Review

Over the years, a number of countries and organizations have embraced sustainability and the circular economy approach. In this section, we will go through literature and mention the different stand out approaches taken, government policies, and the impacts. The two subsections show the road maps taken by the rest of the world and the road map being taken by South Africa.

### The Rest of the World

Since 2010, the Ellen MacArthur Foundation, created by a global yachtswoman, has raised awareness of the concept among manufacturers and policy makers. And since the 1990s, circular economy principles have been actively extended to small scales in eco-industrial parks such as the Kalundborg Symbiosis in Denmark (Stahel 2016). Over the last decade, South Korea, China, and the United States have initiated research projects to promote circular economies by boosting remanufacturing and re-use. Europe is following, but are taking baby’s steps. The Swedish Foundation for Strategic Environmental Analysis (Mistra) and the EU Horizon 2020 initiative released their first call for proposals for a circular economy in 2014. The European Commission proposed the Circular Economy Package to the European Parliament in December 2015.

China and South Korea have run industrial parks for the past 20 years using the principles of the circular economy to link business supply chains and reuse or recycle everyday materials. China has accredited more than 50 parks of this kind. China’s parks saved 14 million tons of GHGE by recycling plastics in 2016, equal to driving more than 3 million vehicles off the track. Atypical park saves more than 2 million tonnes of CO_2_ emissions a year. Countries in the European Union (EU), Japan, the United states (US), Brazil, and India, have adopted similar methodologies. Some researchers claim that industrial parks built for a circular economy may have significant limitations, since the interdependence of suppliers poses a risk. Moreover, these parks cannot be developed just anywhere. Selection of the site depends on the local production goals and the cultural, social, and technical conditions of the region (Miao and Tang 2016).

China, Japan and South Korea have national policies to enable a circular economy. In 2008, China adopted its regulations to eliminate, reuse, and recycle urban waste and manufacturing by-products. The government has spent billions in demonstration projects, introduced tax incentives and released permits that encourage the industry to carry out activities that had previously been prohibited. Activities like these will save Chinese companies and households US$4.6 trillion in 2030, or 14% of their estimated gross domestic product that year. In Kawasaki, Japan, the re-use of industrial and urban waste in cement processing has decreased GHGE by nearly 41,300 tons a year since 2009 and saved 272,000 tons of virgin materials annually.

The EU has introduced binding targets for urban waste. By 2030, Member States must re-use and recycle at least 65% of waste and not send more than 10% to landfill sites. The aim is to ensure that all plastics are reusable and that 75% of packaging is recycled. In the 2018 Eurobarometer poll, 41% of small businesses and 53% of major corporations across the EU reported lowered manufacturing costs by adopting the circular economy approach. In 2011, 24 cement companies in 100 countries announced that they had replaced 13% of their primary fuels with those extracted from waste, lowering CO2 emissions by 17 million tons per year (Geng et al. 2019).

**Fig. 3:**
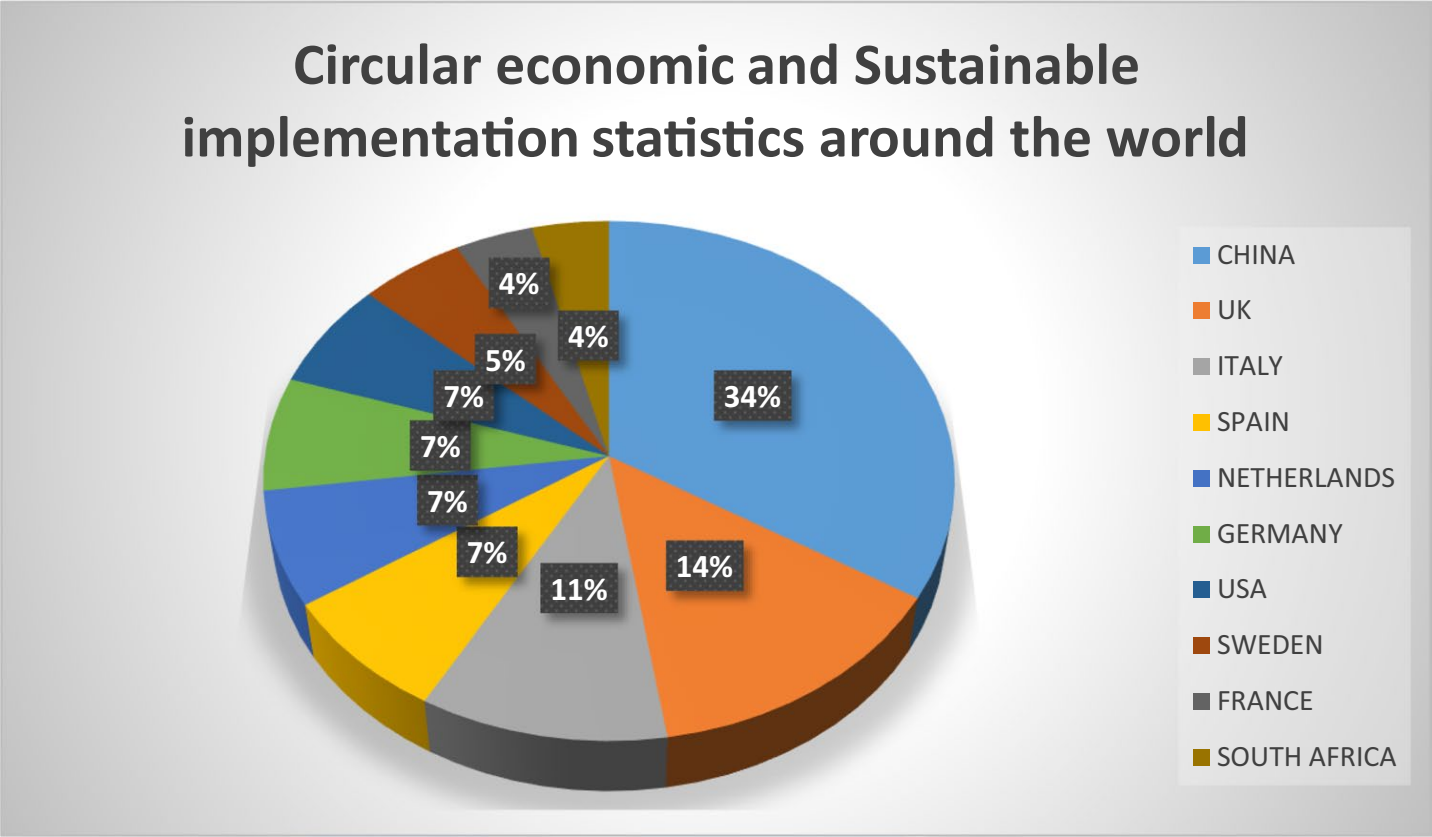
We notice from the stats in the chart that South Africa are still way behind with regard to implementations compared to the likes of China and the EU

China is one of the most studied countries among the globally classified applications. Circular economy is interpreted as the environmental component of China’s overarching vision of a “harmonious society,” conceived by the 16th Party Congress in 2002 in reaction to social and environmental issues following China’s unfettered economic development strategy following Mao Zedong’s death in 1976. The 18th Party Congress of the CCP in 2012 further reinforced its official commitment to establish China as a “ecological civilisation” marked by the unity between man and nature in the “Beautiful China” epitome (Naustdalslid 2014). Increasing government participation in the process of China’s economic transition is taking place, and this involvement also plays a key role in the introduction of the circular economy. While Europe is one of the leading areas in the use of circular economy principles by administrative oversight and policy-making. In addition to China and Europe, many other geographical case studies are available in literature. Research has been done for the water market in Jordan, where there is a great potential value from the implementation of the circular economy. Another researcher looked at improvements in the Serbian waste management system. The Arabian Gulf Region is the biggest source of oil in the world; researches are implementing circular approaches for environmental sustainability (Gulseven and Mostert 2017). In 2016, the government of Taiwan unveiled a range of steps to introduce a circular economy. A total of 66 circular implementations have taken place in Taiwan in the latest publication, involving more than 360 partners. Taiwan being the leading producer and maker of electrical devices, the outstanding program was the establishment of a circular economy for environmental economic development (Ibitz 2020). A similar article to this paper was published recently where researches compared the circular economy roadmap of France and Singapore. Particularly with regard to their waste management system, along with proposals to establish a circular approach in both countries (Ghomi et al. 2021). A visual representation is seen in Fig. 3 of the some of the country’s leading the way towards sustainability and a circular economy (Ngan et al. 2019).

Major Cities drive the world economy. Today, more than 55% of the world’s population lives in cities and is projected to grow to 80% by 2050. City dwellers produce 80% of global GDP. Furthermore, cities as a whole presently need 40 billion tons of energy to sustain their habitats, which is likely to exceed 90 billion tons by 2050. The Asian Development Bank estimates that 21 out of 37 mega cities in the world – cities with 10 million or more inhabitants will likely be based in Asia and the Pacific region in 2025. With the increase in these numbers globally in cities, sustainability and CO2 emissions come in to question, therefore circular approaches could be an ideal solution. Pioneering the path ahead, Singapore is an example of how “circular-to-be” cities are evolving and is thus worth analyzing. Singapore introduced 2019 as the Year for Zero Waste as it is moving towards becoming a Zero Waste Country by reducing its resource use and rising re-use and recycling rates. Three waste sources were targeted: e-waste, packaging waste, and Food and emerging technology and creativity that the government is pursuing to fully close waste loops. A Resource Sustainability Bill has also been published. Once ratified, it will enforce responsibilities pertaining to the storage and disposal of electrical and electronic waste and food waste, to include the reporting of packaging transported into or used in Singapore, to control individuals running producer accountability schemes and to encourage the sustainability of land (Carrière et al. 2020).

As countries around the world struggle with the effects of COVID-19, there is increasing awareness that the conventional linear path to economic development is no longer sustainable. Many analysts agree that the crisis triggered by the coronavirus pandemic is an opportunity to follow a modern solution, and many countries are now taking aggressive steps for the development of a circular economy (South Africa Department of Science and Technology 2020). While countries around the world have adopted the circular approach and have implemented policies, companies around the world have made their own efforts (Hoosain et al. 2020b).

- Timberland—from tires to shoes.
- Johnson controls—recycled batteries.
- Aquazone—water waste in to fertilizer.
- Schneider electric—increase commodity life by leasing and pay for each use.
- Michelin—their systems called EFFIFUEL, Vehicles use the IoT sensor environment to suggest and train eco-driving strategies.
- EON ID—the first RFID tag for the industry in the form of a thread that can be paired with textiles to enable recycling.
- Veolia—Memthane Technology turns 98% of organic runoff into biogas, generating 10% of Mars Netherlands plant capital per year.
- PANGAIA—a sneaker newly introduced, made from recycled grape leather, comes from waste in the Italian wine industry. 26 billion litres of wine are produced annually, with a waste of about 6.5 billion tons.

While companies and organizations around the world are making a substantial effort, it is of utmost importance that there are multidispliniry collaborations. The International Institute for Environmental Studies in Peterborough, Canada, is doing just that. The institute includes 19 universities and research institutes around the world, has set the circular economy as a focus field and encourages joint PhD programs. The importance of the circular economy to environmental science has contributed to a substantial rise in scientific work over the last few years. China, the United Kingdom, Italy, the Netherlands, and Germany became the five most productive nations (Ruiz-Real et al. 2018).

#### The Built Environment

The building sector produces the highest quantity of waste, consumes the most resources which is approximately 20 tons/year, and has a high global effect with regard to its CO2 emissions. By the year 2050, these figures are expected to double. The industry has adopted a number of circular economy concepts globally in order to curb 2050 prediction and align with the SDGs. The circular approach has gained a greater presence in the industry after a series of studies were released by the Ellen MacArthur Foundation. The European construction industry has primarily concentrated on reducing operating energy usage in order to reduce the environmental impacts of buildings. In recent years, the EU’s action plan has a range of actions and policy proposals, including preparations for potential reuse and recycling goals for building and demolition of waste (Eberhardt et al. 2020). We list a few concepts within the built environment below (Finamore n.d.):

- Queen Elizabeth Olympic Park in London—An asset recycling scheme was developed to assist contractors in the re-use of products after the games by selling them or contributing them to charities. Sustainability was an excellent feature, as the local neighborhood benefited from the complex.
- New office building in Essen Germany—Previously, it was an industrial coal mine complex. The effort focused on the environmental nature of the cradle-to-cradle design.
- Liander head office in the Netherlands—It is the first building in the Netherlands to be ecological and to be energy positive. Less materials having been used, re-use of materials will also continue their full life cycle and reduce carbon emissions.
- Lafarge in partnership with UN-SDGs—Developing waste and manufacturing environmental policies in factories focused on a circular economy model, with the potential to allow better use of by-products and waste as raw materials and renewable fuels.

#### Data Centers and Digital Technologies

We live in an era where data centers are increasing, requiring more space, and are a vital part of metropolitan neighbourhoods, using vast quantities of natural resources and being the leading driver of global energy consumption for the foreseeable future. Because of repercussions such as the COVID-19 pandemic, which is changing the need for digital infrastructure throughout the world, this demand is disruptive and at a time of both hazard and opportunity. With the worldwide drive for zero carbon emissions, solutions for data center decarbonization must be implemented. New breakthroughs are made accessible, with economic, social, and environmental implications for data centers. Circular economy and fourth industrial revolution digital technologies are useful procedural tools that can be used to systematically analyze data centers, control their mining and critical raw materials, and aid in the transition to a sustainable and circular data center by objectively assessing the environmental and economic impacts, as well as evaluating alternative options. According to study, reducing the environmental impact and energy usage of data centers is insufficient. Both embodied and operational emissions are crucial in data center architecture. Data centers also play an important societal role in our daily lives, allowing us to freely exchange data and interact via social media, deal on the block chain with cryptocurrencies, provide free online education, and create jobs. As a result, sustainability and efficiency measures, such as circularity and its accompanying tools, as well as newer technology, have developed in a number of ways.

To reach the future efficient, sustainable, and circular data center, we will need to embrace ideas like SustainInfra and SustainTech, as well as firms like Google and Microsoft. We must cut carbon emissions to reduce data center environmental deterioration. Every year, 50 million tonnes of e-waste made up of crucial raw materials that may be reused, reconditioned, or repurposed are discarded. By 2050, this figure is predicted to more than triple to 110 million tonnes. As a result, employing circular techniques for future sustainable data centers would undoubtedly be beneficial, particularly in terms of limiting hazardous and unethical mining and material processing. By adhering to the ground principles established by the UN-SDGs, we may manage emerging technologies, engineering, and data center advances for a better environment and a better society for all (Hoosain et al. 2022).

Globally, there are numerous case studies of the circular economy, with a focus on waste management and innovative economic models but less on social implications. There are many small-scale examples of circular processes in developing economies, such as India and South America, such as waste collection, recycling, repair, and refurbishment. There is a stronger focus in Africa on job growth and on optimizing the usage of capital. However, the circular economy as a term is still obscure in Africa, although there are case studies that have remained largely secretive so far.

#### The BRICS Nations

We must keep in mind that South Africa is a member of the BRICS. The economies of Brazil, Russia, India, China, and South Africa are together referred to as BRICS. Each of the BRICS nations is contributing in their own unique way to the fight against climate change and the fulfilment of the UN-SDGs. According to research, the commitments of Brazil, Russia, India, China, and South Africa to their respective nationally determined contributions (NDC) to the Paris Agreement are currently rated as “insufficient,” “critically insufficient,” “compatible,” “incompatible,” and “highly insufficient,” respectively. As a result, positive effects can be achieved by increasing low-carbon financing and investments, emphasizing taxation that goes beyond energy, investing in low-carbon cities, embracing a circular economy and low-carbon technologies, growing the electricity market, and encouraging climate-friendly trade among the BRICS nations.

The exploration of the substantial prospects linked to the shift to a more circular economy has been spearheaded by China and India, we look at some initiatives of these two nations compared to South Africa in Table 3. Through innovation and early adoption of more sustainable and equitable economic paths, it gives emerging economies the chance to leapfrog the development patterns of the Global North. It also offers a chance to create more resilient, sustainable economies that can better withstand shocks in the future, including pandemics, resource shortages, and climate-related natural disasters like droughts and floods, all of which are expected to become more frequent and severe in the coming years (Sampene et al. 2021).

**Table 3:** Examples of recent CE applications and programs implemented in BRICS countries

Country	Example/description
China	The Work Plan for the Pilot Program of “Zero Waste Cities” was published by the State Council of China on January 21, 2019. This is of tremendous significance for advancing and deepening comprehensive reform of urban solid waste management The 13th Five-Year Plan of China was enacted into law on March 15, 2016. The Plan’s main objectives include encouraging circular production to create a circular economy at all societal levels Government has also created special funding programs for promoting CE and sustainability
India	The Ministry of Environment, Forests, and Climate Change adopted the Extended Producer Responsibility Law on E-Waste in 2016 Maharashtra’s water reuse regulations say that cleaned wastewater can be used for non-potable applications including cooling industrial estates and cooling thermal power plants EU partnerships for further innovation Funding schemes such as Clean India Mission and Switch Asia
South Africa	Government circularity implementations are relatively infrequent. With regard to the Green Economy Strategy Framework, the western cape administration appears to be setting the pace at the present A change in this direction is currently being sought after by the National Government, which is also developing broad National CE standards. Additionally, the Carbon Tax Act, which aims to cut industrial greenhouse gas emissions from significant emitters, has finally become law Compared to their BRICS counterparts, there are not sufficient funding programs

The BRICS governments noted that COVID-19 has increased social vulnerabilities, caused to major job losses, and posed a serious obstacle to fulfilling the ambitions of the 2030 Agenda for Sustainable Development and Sustainable Development Goals during the sixth BRICS Environment Ministers’ Meeting. The main takeaway from the discussion was that the BRICS countries urged national plans for economic recovery following COVID-19 to include actions to enhance the environment and develop the circular economy in the context of sustainable patterns of production and consumption.

### South Africa

The African Circular Economy Alliance, founded by the United Nations Environment Programme (UNEP), includes South Africa as a founding member. South Africa, Rwanda and Nigeria decided to push forward the findings of the African Ministerial Conference on Climate (AMCEN) in cooperation with the World Economic Forum (WEF). The first introduction of circular economy in South African was in the Science, Technology, and Innovation (STI) White Paper of 2019. The paper had defined the circular approach just as the Ellen Macarthur foundation did.

South Africa is pioneering this new circular model as a sustainable development paradigm that has the potential to solve existing environmental and resource conservation issues while at the same time improving resource production and eco-efficiency. Efficient adoption of a circular economy is seen as one direction in which South Africa will progress from its past environmental harm characteristic of emerging economies. As reflected in research, the use of recycled raw materials is comparatively limited and the costs of implementation are a further obstacle in SA (Tahulela and Ballard 2019), while further research has shown that cost saving was considered to be the best catalyst and maintainer for the recycling of materials. Maximizing cost-cutting potential is also a crucial factor in motivating South African businesses to adopt the circular economy (Mativenga et al. 2017).

#### Government Plans and Policies

**Fig. 4:**
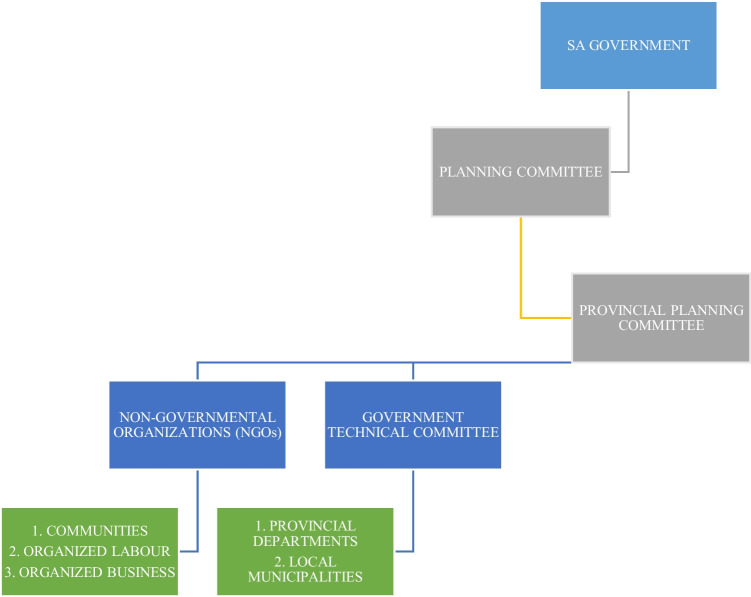
A Tree diagram of who is driving the NDP towards sustainability in SA

The SA government has recently approved the National Waste Management Plan for 2020. The goal of the National Waste Management Policy 2020 is to support the waste hierarchy and the ideals of the circular economy, thus obtaining both socio-economic gains (job creation) and reducing harmful environmental impacts. The policy offers government technical interventions for the waste sector and is compatible and sensitive to the SDGs. This is in line with South Africa’s National Development Plan (NDP), “Vision 2030.” Which is the country’s unique response to Africa Agenda 2063, and the incorporation of the SDGs into their overall socio-economic development strategies. The NDP seeks to eradicate hunger and reduce inequalities by 2030. According to the program, SA will accomplish these objectives by drawing on the energy of its population, by creating an inclusive economy, by building infrastructure, by improving state capacity, and by fostering leadership and alliances through society. We graphically show who is responsible for driving the vision within the country in Fig. 4 (Subban and Theron 2016). The country is made up of 9 provinces with 3 capital cities; provinces have drawn up their own plans to align themselves with the NDP (South Africa Government n.d.). We analyze some of the key provinces plan in Table 4, particularly the ones linked to a circular economy and the SDGs (South Africa Department of Environment, Forestry and Fisheries 2020).

**Table 4:** Individual key state plans

Province	Plan
Eastern Cape	The Eastern Capes plan is to spread creativity and manufacturing, and people who can support themselves. Both children and youth express the common conviction that they are the foundation of the future. Participatory engagement on local growth guided by dedicated, competent people and responsible institutional agents (Eastern Cape planning commission 2015)
Gauteng	The main emphasis will be on the economy; employment and infrastructure; education; the growth of skills and health; organized human settlements and land release; defence, social stability and food security as well as creating a capable, ethical and developmental state. There will be ambitious measures to deepen the introduction of the Transformation, Modernisation, and Re-industrialisation program. 4IR; Finance and Retail; Tourism and Hospitality; Creative and Cultural Industries; Manufacturing and Warehousing; and Green Economy will all be core sectors of focus (News SA 2021)
KwaZulu—Natal	Known as the Provincial Growth and Development Plan. Priorities include providing more jobs, good employment and sustainable livelihoods for economic growth; rural development, land reform and food security; enhancing basic education quality; long and safe living for all South Africans; and reducing violence and corruption
Limpopo	The Limpopo Provincial Government is in the process of updating and aligning its growth and development policy with the NDP to place the province on a higher economic development path. Limpopo’s industrial growth and prosperity was rooted in the three main comparative advantages of mining, agriculture and tourism. The province is also rich in mineral deposits such as silver, chromium, coal and diamonds (South African Government News Agency 2014)
Western Cape	The Western Cape has identified five Vision-inspired Priorities (VIPs) that measure their contribution to finding opportunities to better the lives, livelihoods and experiences of people. This Provincial Strategic Plan outlines how: (i) developing healthy and inclusive communities; (ii) improving the economy and generating jobs; (iii) inspiring our people; (iv) fostering mobility and urban transformation; and, at the same time, (v) pushing creativity through the community (Western Cape Government 2019)

#### Organizations and Government Projects and Applications

To date many organizations within the country has responded to the circular approach, because it assists with sustainability as well as create newer revenues and business models.

- The Western Cape Industrial Symbiosis Programme (WISP) is an award winning organization that is funded by government. This organization has assisted many businesses in reducing their wastage using circular approaches, while at the same time improve profitability.
- AB-InBev, COKE, PETCO, SAB are all major companies that have adopted a circular economy. They have increase recyclability with returnable plastic and glass bottles.
- SPACE Project-Water and waste management technologies in informal Langrug Settlement using the concepts of Biomimicry to clean rain water, flood water, and industrial waste.
- Barloworld—Caterpillar parts are now repaired and refurbished with a new guarantee (Desmond and Asamba 2019).
- Design futures Africa is about circular designers in Africa, the featured projects and designers were hosted and supported by the British council and the Ellen MacArthur foundation at the Circular Design Lab in 2019. The projects can be found here www.twyg.co.za.
- The Recycling and Economic Development Initiative of SA (REDISA) is a South African NPC that works with governments to implement circular economies. They have reshaped the tyre recycling value chain with one of their projects.

**Fig. 5:**
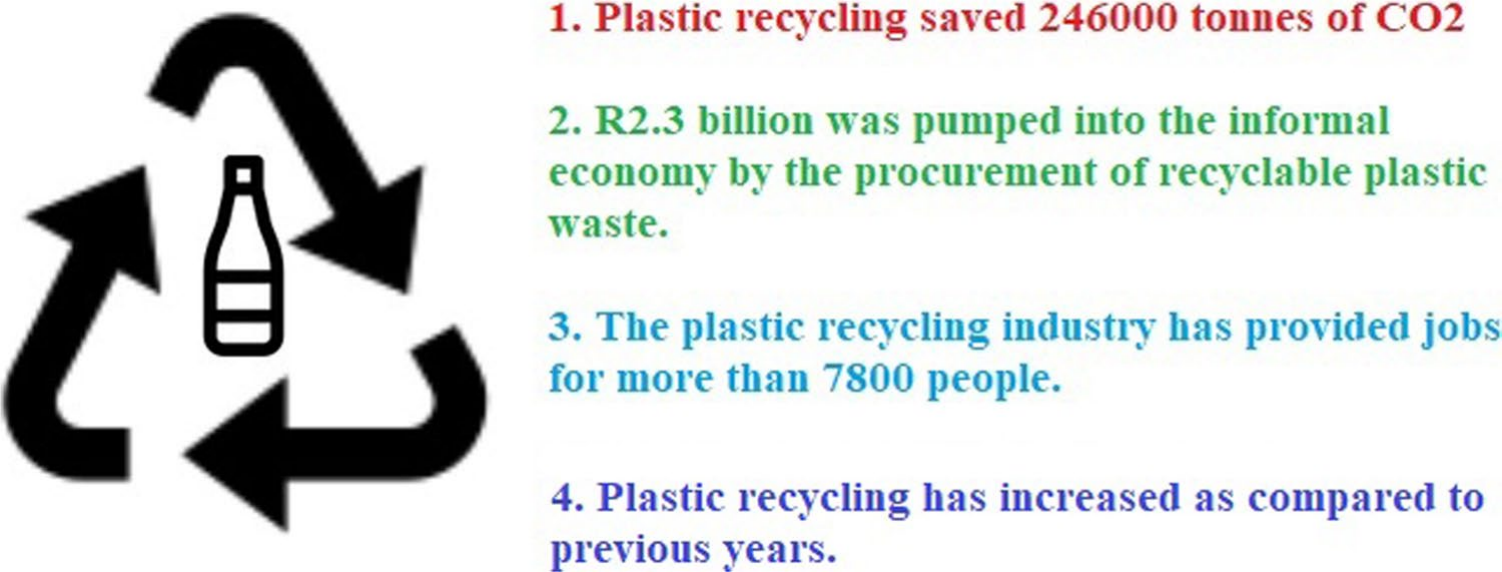
The impact of plastic recycling in South Africa (FASA n.d.)

Recycling stats show that South Africa is playing its part according to the Franchise Association of South Africa which is shown in Fig. 5. Plastic Pacts is the New Plastics Economy and has set out a vision for a global plastics system in which plastics never become waste—a circular economy for plastics. In 2020, The Ellen MacArthur Foundation welcomed the announcement of the South African Plastics Pact, the first on the African continent to join the global Plastics Pact network. The South African Plastics Pact founding members include some major partnerships: The Clicks Group, Coca-Cola Africa, Danone, Distell, Home Choice, Massmart, Myplas, Nampak Rigids, Pick n Pay, Polyoak, Polyplank, Shoprite Group, SPAR, Spur Holdings, The Foschini Group, Tiger brands, Tuffy, Unilever, ADDIS, Waste Plan, and Woolworths. Supporting members include industry associations Fruit South Africa, The South African Plastics Recycling Organisation, The Polyolefin Responsibility Organisation NPC, the Polystyrene Association of South Africa, the PET Recycling Company, the Southern African Vinyl Association, and The Institute of Waste Management of Southern Africa. As well as the National Department of Environment, Forestry and Fisheries (DEFF) and the City of Cape Town (SA Plastics Pact 2023).

A number of departments within government have implemented policies towards a circular economy. One example is, The Scientific and Industrial Research Council (CSIR) has been appointed by the Department of Science and Technology (DST) to enforce the 10-year Waste Research, Development and Innovation (RDI) Roadmap for South Africa. This roadmap covers the prevention of waste and the optimized extraction of value from reuse, recycle and regeneration, with a view to producing substantial social, economic and environmental benefits through investment in research, growth and innovation. One of the five priority waste sources to be tackled is waste electronic and electrical equipment (WEEE). Other noticeable changes include; Carbon Tax act, Plastic taxes and levy’s, waste levy’s, and sin-taxes to control consumer behaviors. The policies and reports can be found here (Voluntary National Review 2019; Lydall et al. 2017).

Department of Environmental Affairs together with the Department of Planning, Monitoring and Evaluation initiated a Chemicals and Waste Phakisa program from 24 July to 24 August 2017. Chemicals and Waste Phakisa’s goal was to invest in opportunities that would lead to a decrease in environmental effects while rising the contribution of GDP and generating jobs. 4 waste streams were identified namely, Municipal Waste, Bulk Industrial waste, Product Design and Waste Minimization and Chemicals. Three feet plans and proposals for budgets with implementation strategies were devised as follows (i) reduce harmful environmental and health effects of waste and threats posed by chemicals, (ii) increase the commercialization of the circular economy and generate value from services that are actually discarded as waste, and (iii) promote balanced development by positioning South Africa as an internationally competitive producer of sustainable goods ( Department of Environmental Affairs and the Department of Planning, Monitoring and Evaluation 2017).

**Fig. 6:**
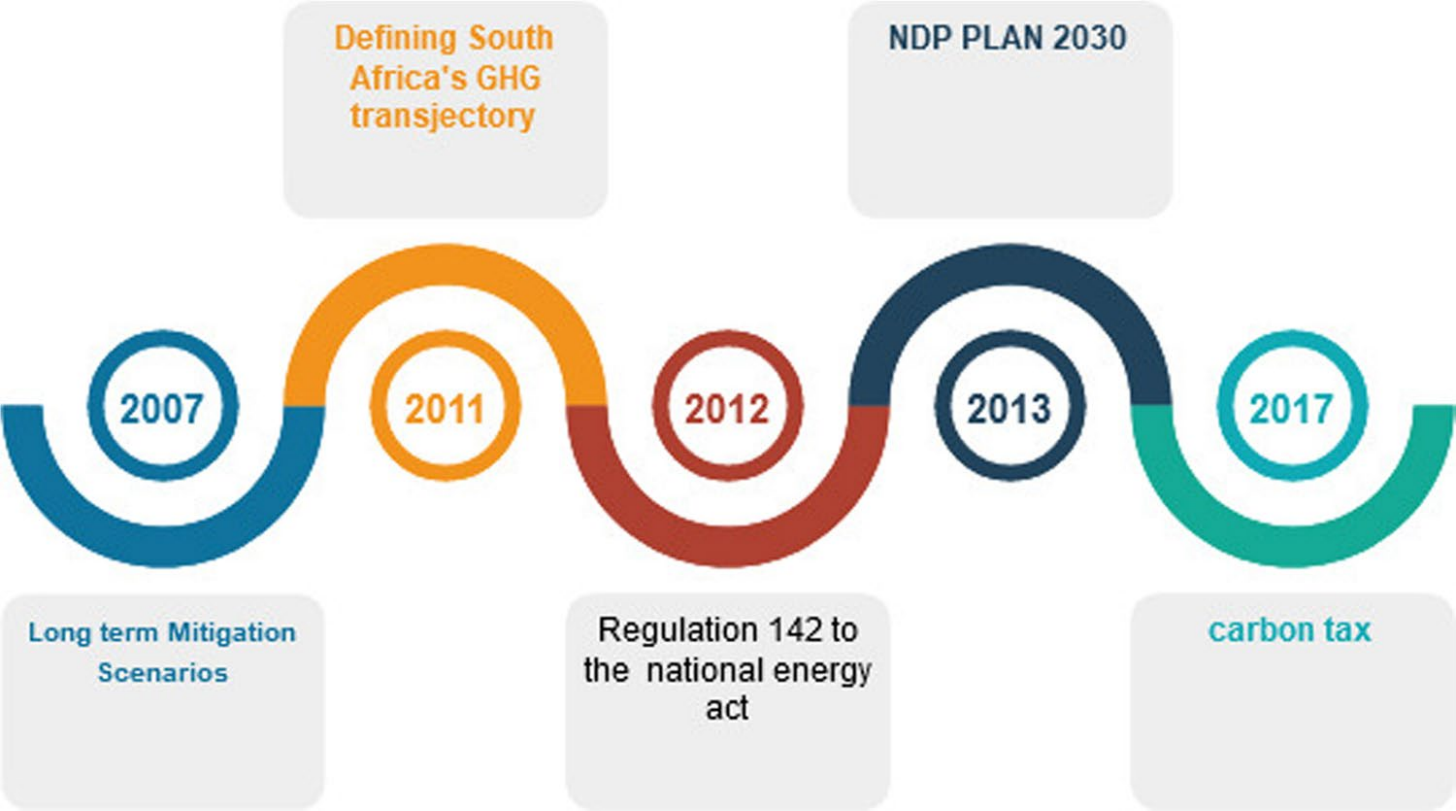
South Africa’s efforts towards a low carbon economy

The United Nations Framework Convention on Climate Change (UNFCCC CoP21) 21st session, which took place in Paris from 30 November to 13 December 2015, resulted in the adoption of the Paris Agreement. The Agreement was implemented after four years of intensive talks required by the 17th UNFCCC CoP held in Durban, South Africa in 2011. The Agreement is a holistic mechanism that will direct international attempts to minimize greenhouse gas emissions and to resolve all related climate change challenges. The Minister of Environmental Affairs, Mrs Edna Molewa, had signed the Paris Agreement on climate change at the United Nations in New York. South Africa is already working on climate change. The nation has made substantial investments in solar energies, mass transport, energy conservation, waste management, and land preservation initiatives. South Africa is also working to improve attempts to shift towards a lower carbon economy and community, as well as to respond in the short-, medium-, and long-term to the impacts of rising temperatures and declining rainfall in many areas of the world (Department of Environment, Forestry and Fisheries of the Republic of South Africa 2016). The incremental reorientation of the world economy towards putting the concept of environmental growth into line has introduced new ideas on how businesses handle problems relating to toxic greenhouse emissions that contribute to global warming and, in turn, climate change. South Africa’s position on carbon emissions has improved over the last few years; this timeline can be seen in Fig. 6 (Bimha and Nhamo 2017).

#### The Mining Sectors

Mining is a huge industry sector in South Africa and the African continent as mineral resources are abundant. Therefore, circular approaches to preserve resources would be a huge step forward. Numerous mines in South Africa already use some aspects of a circular economy, and they have the ability to expand their efforts to have a significant impact on the economy.

In order to extract ores with more precision and efficiency while using less energy, water, and capital, and producing less waste, new innovative technologies are being investigated (Godfrey 2021). Research has been done for the gold mining industry and the implantation of a circular economy. There were 5 key points that stood out: facilitating system effectiveness; preserving and enhancing renewable resources; optimizing resource yields; collaboration, and enhanced business models which entail transparent regulatory reporting (Parker 2019).

#### The Farming Sectors

In South Africa farming is vital with respect to its export and local markets. A wide range of policies that apply to agriculture and agro-processing are available in South Africa. But, climate change has a direct impact on agricultural productivity. Household and small-scale farmers are restricted by inadequate infrastructure and logistics. Lack of access or education to newer technologies, such as mobile or digital platforms or technologies for precision farming, frequently limits opportunities to address some of these issues.

The water-energy-food Nexus (FEW) is essential to South Africa’s economy, and any rise in demand in one area has a ripple impact on the others. For South Africa, changing to a more circular agriculture system has tangible positive social, economic, and environmental effects. To enhance sustainability and open up new economic prospects, the industry must change from a linear to a circular one. We believe this can be done using new technologies provided by the 4IR which can lead to Smart Farming. Furthermore, research has shown that the circular economy and 4IR technologies have a positive influence on the Climate–Water–Energy–Food Nexus and the SDGs (Hoosain et al. 2023). Improved food security, resilience, global competitiveness, economic growth, job creation, decarbonization of the sector, sustainable resource utilization, and sustainable food systems are some benefits of this transition and adoption of new technologies, which are all in line with the SDGs (Godfrey 2021).

## Solutions and Guidelines

After going through literature, we had found that there are major efforts being taken globally towards sustainability and transitioning towards a circular economy. Most of these efforts seem to be much stronger in the east and the European countries. Governments are working fast to introduce strategies on the circular economy, like ESG reporting and others. Bloomberg Greens latest study covers circular economy strategies in 26 markets, which collectively account for 88% of world GDP. Increasing globalization ensures that circular economic strategies have an impact not only on material sustainability but also on foreign trade flows (Savut 2020). We had only mentioned a handful of countries in this paper, many more global implementations, best practices, government interventions, barriers and solutions, and replacement of a linear economy can be found here (Ghosh 2020).

**Fig. 7:**
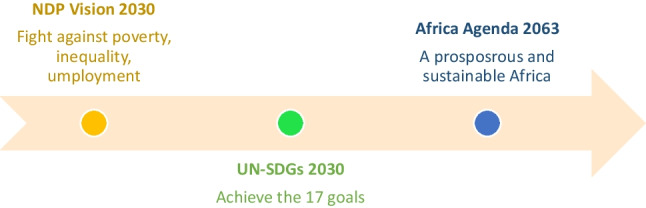
South Africa’s goals and vision for the future

After analyzing the 2019 Voluntary National review with the UN, the National Planning Commission of SA and Stats SA, it was found that South Africa had made significant strides thus far:

- Achieving the SDGs is in South Africa’s primary interest.
- Significant progress has been made since democracy in 1994, with improved living conditions for millions.
- Access to clean water, energy, sanitation, health, and education.
- Gender equality is on the rise, for example, women in key positions in parliament has risen.
- As per the Paris agreement investments have been made towards climate change, renewable energy, waste management, and land restoration.
- Continuing investment in science and technology. During his 2018 State of the Country speech, the President of the Republic of South Africa called for the creation of a Presidential Commission on 4IR to ensure that South Africa is in a position to capture the resources and manage the complexities of rapid technological advances.
- Finally, SA has plans in place and this is shown in Fig. 7.

Although these strides are a great step forward, there are still major challenges as described in the report and by the national Statistics (Department of Environmental Affairs Republic of South Africa 2019; South Africa Department of Statistics 2020). South Africa and/or the African continent for that matter, have put policies in place, and their efforts are quite innovative, they are unfortunately sporadic. This comes down to a number of factors; the funding towards this innovative global approach is much less compared to the first world countries, the public mind-set is quite different, corruption, and criminal activities, and education seems to be lagging. The stand out point that was mentioned and is in direct relation to this paper is SA’s transition towards a circular economy, reduction in carbon emissions as the reliance fossil fuels are still high. In the next subsection we will suggest solutions for the future of South Africa’s sustainability and transition to a circular economy by following the road map taken by their global counterparts.

In order for South Africa to break the barriers towards sustainability and a circular economy, it needs to follow the road maps taken by their global counterparts. We suggest some solutions below:

- The key player to accelerate the process is, government interventions. Stricter policies need to be enforced, and monitored on a regular basis.
- Companies need to be incentivized as they are afraid of the costs that will be incurred during the transition process. Similarly, with government interventions, government capital support and incentives towards companies can assist the process.
- Education is of utmost importance. The public need to break the cultural barriers and mind sets, and make the necessary changes to their lifestyles. Research was done recently between oxford university and the UN, they had conducted a global climate poll. It was found that two thirds of people globally had said that climate change is an emergency. The key point in this poll was that the two thirds who have called for action are mainly from higher educated nations (Peoples’ Climate Vote - Results 2021). In 2019, a young Swedish teen, Greta Thunberg became Time’s Person of the Year after she vowed to take a stand and convince the world that her generation, Generation Z, would no longer accept the degradation of the planet.
- Resource prices and allocations need to be controlled, so as to force circular thinking.
- Waste management and recycling need to be further addressed and monitored. Fines can be put in place for mismanagement.
- Partnerships among the private, public, and governmental organizations are of utmost importance.
- With the use and acceptance of newer 4IR technologies, databases should be created in order to monitor the countries resources and life cycles. Digital marking of materials using AI and IoT, and material passports are a great solution towards tracking life cycles of materials. This should be a part of a global effort, so that countries can work together.
- ESG reporting and investments need to be made mandatory.
- Public transport is South Africa is based mainly on Taxis. Therefore, the population relies on their own cars. With this comes huge CO2 emissions. There either needs to be stricter Laws put in place to reduce emissions, fix the public transport or embrace the newer electric motors.
- Key performance indicators (KPIs) should be created through the International Organization for Standardization (ISO).
- Major finance allocations need to be put in place with stricter spending measures (no corruption), specifically for the transition to a circular economy and achieving the SDGs.
- Lastly, South Africa’s main source of energy comes from the local energy supplier (ESKOM). Unfortunately, fossil fuels are a huge source for this energy. Recently, the country has been battling with load shedding due to mismanagement, lack of maintenance and theft. Given the challenges and the problems, this is the perfect time for government to invest in renewable sources, while at the same time investing in climate friendly measures and transitioning to a circular economy as this will positively affect all sectors.

## Discussion and Conclusion

In this paper, we had compared South Africa’s sustainability and circular economic road map to the rest of the world, so that suggested solutions and policies can be put in place for the future of the country. We had explored many sustainable efforts and circular initiatives around the world through literature, government publications and statistical reports. Yes, countries around the world have their challenges, but they have made much more progress compared to South Africa. Although sustainability and the circular economy narrative are other countries, specifically in Europe focuses on economic and environmental issues with less emphasis on social effects.

South Africa’s economy is plagued by poverty and inequality, high levels of unemployment, a carbon-intensive sector, water scarcity, and sluggish GDP growth. South Africa has made strides towards sustainability and the transition to a circular economy in the form of their NDP Vision 2030 as well as achieving the UN-SDGs, but they are not nearly enough. The government believes that the disruption caused by the COVID-19 pandemic allows them to usher in the necessary policy changes linked to Extended Producer Responsibility (EPR) policy, including establishing goals for product composition that would spur a green economic rebound and put SA on the path towards a circular economy. We believe that this is not the case, rather the COVID-19 pandemic has hampered/delayed South Africa’s future Vision as the economy along with many other developing countries have taken a huge knock. We had looked at the numerous challenges affecting SA based on the national stats, and we suggested a number of solutions for the future. Some of which include; the transition towards a circular economy in South Africa can be a solution towards achieving the UN-SDGs. Concepts such as ESG reporting and 4IR digital technologies can assist in the transition as the positive effects globally have been seen. Education towards sustainability needs to be improved throughout all sectors, with further assistance from government with respect to stricter policies and financial assistance or incentives for businesses. Importantly, coal supplies roughly 72.1% of the nation’s basic energy requirements. While at the same time, they experience serious energy problems. Future plans must include the usage of renewable energy sources and a government multidimensional energy action plan with the goal of ensuring South Africa’s energy security in the near future.

It is clear to say, that yes there are obstacles to creating a sustainable and circular economy internationally and locally, but with certain long-term plans, the idea of being a sustainable and circular world will become a reality through global partnerships, education, and public and private compliance. Global initiatives such as the decoupling of economic development, the reduction of carbon and the transition to a renewable production chain are crucial factors for progressing towards sustainability and a circular economy. It is also important to evaluate the effect of national plans and scenarios in a global context, not just a single region. As the world slept during COVID-19 lockdowns, the environment had blossomed. The question remains, have we made changes for a better world, post COVID.

## Data Availability

All data generated or analyzed during this study are included in this published article.
